# Mr. Wainwright, on Vaccination

**Published:** 1805-11-01

**Authors:** Thomas Wainwright

**Affiliations:** Dudley


### To the Editors of the Medical and Physical Journal.

GENTLEMEN,

IT seems that the progress of vaccination is more likely to be impeded by the indiscreet efforts of some of its zealous admirers, than by all the hostile attacks of its decided enemies. An instance of the indiscretion alluded to, occurs in the Report made by Mr. Addington to the Jennerian Society, of the progress and success of the vaccine inoculation at Dudley. The Report has also been published in the Gentleman's Magazine for last June; several of this Gentlemen's statements on the subject are incorrect. The enemies to the inoculation for the cow-pock, may, from hence, take occasion to assert, that the misrepresentation was necessary to the credit of the vaccine cause; on the contrary, a more correct, and more faithful account, will be found most honourable and most creditable to the vaccine disease; in as much, as it is of the highest importance to know, that of the four or five thousand persons vaccinated at Dudley, not one has caught the small-pox, though all of them have been, more or less, in the way of infection, and many of them have lived in the same house, and slept in the same bed with persons labouring under that dreadful disease. "Mr. Addington tells the Jennerian Society, that the extirpation of the small-pox, from the populous town of Dudley, must be attributed, principally, to the unremitting zeal of a learned divine, who had early directed the attention of his numerous parishioners to the preventive system of vaccination, and had taken every opportunity of conferring with his medical friends in the place, and of keeping up, in every possible way, the attention of the inhabitants to the subject."

I should be most happy to confirm the assertion, that the small-pox was extirpated, not only from Dudley, but from the face of the whole earth. But unhappily, in May last, the small-pox was in this town and neighbourhood, and continues, at this moment, to be very frequent and very fatal: Seven victims to the loathsome disease were buried in our parish on the 7th, and four more on the 14th of this month. Why does Mr. A. so studiously suppress all notice of the credit due to the patriotic exertions of the medical men in this town, who have devoted their time and talents to a gratuitous vaccination of the poor? Why does he insinuate that the exertions of the medical vaccinators have originated from the influence and persuasion of a much esteemed clergyman? These misreprentations and unjust insinuations, can no way be reconciled with that candour and liberality, which in this country are very generally the concomitants of the medical profession. Surely, it would have been prudent in Mr. A. to have had some communication with the medical inoculators before he presumed to obtrude himself with his report upon so respectable a society. The health of a man is infinitely too important a subject to be trifled with; and, it is most essential to the progress of medical science, that in every case, the truth, the whole truth, and nothing but the truth, be published. If some of these reflections be thought harsh and severe, let every allowance be made for the feelings of a son, indignant at the artful attempt to confer upon another man, that credit which is most justly due to the unwearied and skilful exertions of his father, and of some other medical inhabitants.

The lower class in this town continued to be obstinately prejudiced against vaccination, till they had the strongest proofs of the preventive powers of the cow-pock, to resist the contagion of the small-pox. For in the neighbouring parish of Kingswinford, the numerous workmen of a much loved nobleman, were induced in 1801, by the influence of his lordship's venerated name, to submit their children to vaccine inoculation; and, soon afterwards, a few of his lordship's labourers, resident in the town of Dudley, followed the example. The cow-pock did not become popular in our town, till some of the individuals, vaccinated in 1801, had been frequently exposed, without risk or danger, to all the contagious powers of the small-pox. Those parents who had expeiienced in their own families the preventive efficacy of the cow-pock in 1801 and 2, were the first to bring their younger children to the general vaccination in 1804. Their example, and their loud praises in honour of the cow-pock, removed many of the prejudices of the Dudley people.

The writer of this article, has, under the direction, and with the able assistance of his father, vaccinated three thousand persons, whose names and places of abode were carefully entered in a book, and in which was also carefully noted, all anomalous symptoms as they appeared. But this was not done, as Mr. A's Report states, without inconvenience convenience convenience to any person. For, in our hands, the cow-pock has always been an inconvenience, and, too often, it has proved a serious evil. The system of vaccination, is, in good truth, only justifiable on these strong grounds, that the cow-pock is, upon the whole, infinitely, in every way, a less evil than the inoculated small-pox. Of these three thousand vaccinated persons, all have done well; and though many of them have frequently been in actual contact with the persons afflicted with the small-pox, in its most malignant form, yet no one has caught that dreadful disorder. The lower class are now aware, that when a vaccinated person has (to use their strong expression) the small-pox in his blood, the preventive powers of the cow-pock are not to be depended upon, and in that case, the small-pox will go through its usual regular stages, but yet, the vaccinated part will frequently assume that form and appearance which is characteristic of the vaccine pustule. There were great odds, that out of three thousand children, taken indiscriminately from the general mass of population, that one, at least, would be cut off during the period of vaccination from diseases totally independent of the cow-pock. For though the vaccine disease is a safe and secure protection against the small-pox, yet it cannot be expected to shield the patient from all the disorders to which the human frame is obnoxious.

And many of these children were, at the time of inoculation, affected with various disorders; as dentition, hooping-cough, worms, disordered bowels, &c. all knowledge of which was carefully concealed by their parents or friends, who brought them to be vaccinated.

Various kinds of obstinate cutaneous eruptions are not unfrequently consequent to the vaccination of young children; but they very rarely take place in those who have the cow-pock at the age of ten years, or at any later period.

The vaccine disease has not introduced any new species of cutaneous eruptions, but it is sometimes the exciting cause which brings eruptive diseases into action, which existed before our knowledge of the cow-pock. Hence, we have been induced to give after vaccination, and with great advantage, small doses of calomel and purgatives, as was the usual practice after the small-pox. Since the adoption of this plan, there has been few instances of eruptions from this cause. In consequence of tight bandages on the arm, and other negligent conduct of their friends, there have been several cases of most formidable ulcerations on the vaccinated part, attended with considerable swelling of the limb and extensive erysipelas. When these symptoms were not removed by the application of the extract of lead, they were easily cured by regular dressing of the part affected with the ung. litharg. c. acet. In these cases, mild poultices are of no service, and it is difficult to conceive that it can ever be beneficial, or requisite, to apply caustic to this ulcer. In two cases, from the same misconduct of the parents, an abcess formed in the axilla, which was readily cured by the usual treatment. In an infant of six months, convulsions took place on the eighth day of vaccination, they recurred three or four times at irregular periods, and finally left the child in a tolerable good state of health, and in perfect possession of his faculties. There has been no case, to which the term, spurious vaccine pustule, could with propriety be exclusively restricted, for very anomalous appearances will take place in the vaccinated part, according to the properties of the virus inserted, yet they always differ in toto from the genuine vaccine pustule. If there be sores, or loss of skin, in any part of the body, it is not uncommon for vaccinated children to excite the vaccine inflammation in such parts, by scratching them when their fingers are loaded with the cow-pock fluid. We have seen no case where the vaccine disease could be charged with predisposing to scrophula, which is too frequently the case with the inoculated small-pox.

This town will afford another example to those already recorded in your Journal, of a person being reinfected with the small-pox. A child of Mr. Downing, then resident at Stourbridge, was inoculated in the spring of 1804, by an eminent surgeon of that place, for the small-pox, which the child had in a very favourable way. In May last this child caught the Small-pox and died. In the second and fatal attack of the small-pox, the attendance of the surgeon, who inoculated the child in 1804, was required. He well recollected every circumstance that attended the inoculation of the child, and declares that inoculation and subsequent variolous disease, to have been perfectly regular, and such as he should, in all cases, have relied upon with implicit confidence.

I am, &c.

THOMAS WAINWRIGHT.

